# Development of a serum-free medium for *in vitro *expansion of human cytotoxic T lymphocytes using a statistical design

**DOI:** 10.1186/1472-6750-10-70

**Published:** 2010-09-21

**Authors:** Min Kyoung Jeon, Jong-Baeck Lim, Gyun Min Lee

**Affiliations:** 1 Department of Biological Sciences and Graduate School of Nanoscience & Technology (WCU), KAIST, 335 Gwahangno, Yuseong-gu, Daejon, 305-701, Korea; 2 Department of Laboratory Medicine, Yonsei University College of Medicine, Seoul, Korea

## Abstract

**Background:**

Serum-containing medium (SCM), which has a number of poorly defined components with varying concentrations, hampers standardization of lymphocyte cultures. In order to develop a serum-free medium (SFM) for the expansion of human lymphocytes from peripheral blood mononuclear cells (PBMCs), a statistical optimization approach based on a fractional factorial method and a response surface method was adopted. A basal medium was prepared by supplementing RPMI1640 medium with insulin, albumin, ferric citrate, ethanolamine, fatty acids, glutamine, sodium pyruvate, 2-mercaptoethanol, 1-thioglycerol, nonessential amino acids, and vitamins. We identified additional positive determinants and their optimal concentrations for cell growth through a statistical analysis.

**Results:**

From a statistical analysis using the fractional factorial method, cholesterol and polyamine supplement were identified as positive determinants for cell growth. Their optimal concentrations were determined by the response surface method. The maximum viable cell concentration in the developed SFM was enhanced by more than 1.5-fold when compared to that in RPMI1640 supplemented with 10% fetal bovine serum (FBS). Furthermore, a cytotoxicity assay and an enzyme-linked immunospot assay revealed that the effector function of cytotoxic T lymphocytes generated from PBMCs grown in SFM, by stimulation of peptide-presenting dendritic cells, was retained or even better than that in SCM.

**Conclusions:**

The use of a developed SFM with cholesterol and polyamine supplement for human lymphocyte culture resulted in better growth without loss of cellular function when compared to SCM.

## Background

Adoptive cell therapy using cytotoxic T lymphocytes (CTLs) has emerged as a new approach to treat patients with various types of cancers and viral infections, and its effectiveness has been demonstrated in Phase I/II studies [1-3]. CTLs play an important role in controlling viral infection and eliminating cells with malignant potential. In clinical trials involving the adoptive transfer of antigen-specific CTLs, CTL doses of 10^7^-10^9 ^cells per kilogram of body mass are required to achieve efficacy [4,5]. Thus, there has been considerable interest in developing an *in vitro *system to expand human CTLs for use in the implementation of adoptive immune therapies.

In general, the culture medium for *in vitro *expansion of CTLs is supplemented with serum, usually of human or fetal calf origin [6-8]. The serum supplement, however, significantly affects experimental results, because a large number of poorly defined components including growth factors, antibodies, and other immunologically active substances vary in concentration between batches [8]. Accordingly, a serum-free medium (SFM) needs to be developed for standardization of *in vitro *expansion of CTLs. However, despite the importance of SFM, its development for *in vitro *expansion of CTLs has not been fully substantiated.

Based on the findings of previous studies of SFM for human lymphocytes [8-10], we prepared a basal SFM for human CTLs through several culture experiments. To achieve better cell growth, growth-enhancing candidates lacking basal SFM for CTLs were identified by a literature search. Cholesterol, phospholipids, and polyamines, which are principal components in serum, were selected on the basis of their positive roles in cell growth of many mammalian cell lines [11-13]. Antioxidants, which are known to exert a synergistic effect on cell growth with polyamines [14], were also included as candidates. In order to assess the effects of these candidate components on the growth of CTLs, a fractional factorial design was employed to screen active factors for cell growth, followed by response surface designs to optimize their concentration.

The cellular functions of CTLs grown in the newly developed SFM were also characterized by a cytotoxicity assay and an enzyme-linked immunospot (ELISpot) assay. To generate antigen-specific CTLs, we used cytomegalovirus (CMV) peptide epitope NLVPMVATV as an antigen. Derived from the immunodominant CMV matrix protein pp65, it is one of the most widely studied antigens in clinical studies [15-17].

## Results

### SFM designed by a fractional factorial method

The basal SFM for *in vitro *expansion of T lymphocytes, the components of which are listed in Table 1, was formulated through a literature search and confirmed via culture experiments (data not shown). In order to further improve the SFM, 4 supplements, phosphatidylcholine, polyamine supplement, antioxidant supplement, and cholesterol, were selected as potential growth enhancers, based on their growth promoting abilities reported in previous studies with other cells. Due to a limited cell number, a statistical approach based on a fractional factorial design was applied for efficient testing of selected active supplements. As shown in the matrix presented in Table 2, kinds 8 of SFM were prepared. The first row of (-) elements in Table 2 is a basic assembly referring to the basal SFM. Since the culture performance of PBMCs in these SFM may depend on the donors, three sets of experiments were carried out independently with PBMCs prepared from three different donors. Cell cultures were performed with IL-2 supplementation, as described in the Materials and Methods section.

**Table 1:** Composition of the basal SFM

RPMI1640 supplemented with:	
**Components**	**Concentration (/l)**

Bovine serum albumin	2.5 g
Insulin	5 mg
Ferric citrate	2 mg
Ethanolamine	1.22 mg
Linoleic acid	1 mg
Oleic acid	1 mg
Palmitic acid	1 mg
L-glutamine	584 mg
Sodium pyruvate	110 mg
2-mercaptoethanol	0.78 mg
1-thioglycerol	5.41 mg
RPMI1640 nonessential amino acids	20 ml
RPMI1640 vitamins solution	10 ml

**Table 2:** Matrix of the fractional factorial design (2^4-^^1^) for the 4 supplements.

	Basal SFM supplemented with:			
	
SFM	A	B	C	D = ABC
	
	Phosphatidyl choline (5 mg/l)	Polyamine supplement (1×)	Antioxidant supplement (1×)	Cholesterol (4 mg/l)
#1	-	-	-	-
#2	+	-	-	+
#3	-	+	-	+
#4	+	+	-	-
#5	-	-	+	+
#6	+	-	+	-
#7	-	+	+	-
#8	+	+	+	+

(-) no addition; (+) addition of the indicated amount of additives.

**Figure 1:**
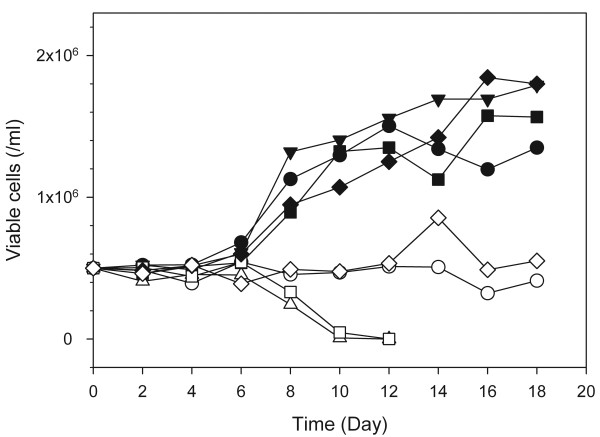
**Growth profiles of cells cultured in 8 different SFM defined by the fractional factorial design**. Cell cultures were replicated independently with PBMCs prepared from three different donors. This figure shows one representative growth profile among the three cultures. Black circles, SFM #1; white circles, SFM #2; inverted black triangles, SFM #3; white triangles, SFM #4; black squares, SFM #5; white squares, SFM #6; black diamonds, SFM #7; white diamonds, SFM #8. The compositions of the supplements for the eight media are shown in Table 2.

Figure 1 shows the growth profiles of PBMCs from one donor (Set #1 in Table 3) in these media with IL-2 supplementation during cultures. Cells cultured in even numbers of SFM (#2, #4, #6, and #8), which are denoted with opened symbols in Figure 1, did not grow well. They maintained their initial seeding density or died gradually. Because the common supplement in the even numbers of SFM was phosphatidylcholine, it is likely that phosphatidylcholine inhibits cell growth. Maximum viable cell concentrations achieved in SFM #3, #5, and #7 were comparable to or higher than that in the basal SFM (SFM #1). Although the growth patterns of PBMCs in the SFM depended on the donors, the general tendency regarding the effect of each supplement on growth did not change significantly (data not shown). The maximum viable cell concentrations achieved in the cultures of PBMCs from the three different donors with the SFM are summarized in Table 3.

**Table 3:** Maximum viable cell concentrations in 8 SFM designed by the fractional factorial method.

SFM	Maximum viable cell concentration (10^5 ^cells/ml)		
	
	Set #1	Set #2	Set #3
SFM #1	15.03	25.38	39.60
SFM #2	5.12	7.83	7.95
SFM #3	17.91	24.84	49.32
SFM #4	4.56	4.62	7.02
SFM #5	15.75	28.80	37.80
SFM #6	5.42	4.72	6.52
SFM #7	18.45	19.44	42.66
SFM #8	8.55	6.03	4.89

PBMCs were obtained from three different donors (Set #1, Set #2, and Set #3). The seeding density was 5 × 10^5 ^cells/ml.

To determine positive factors for cell growth from among the 4 supplements tested, the maximum viable cell concentrations in each culture were evaluated in the normal probability plot.

**Figure 2:**
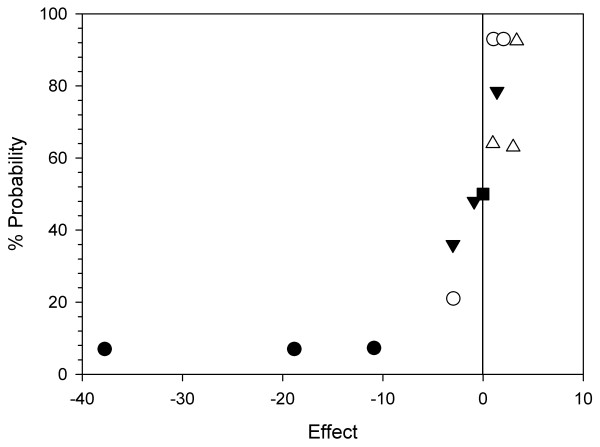
**Normal probability plot of the effects obtained from 4 supplements**. Black circles, phosphatidylcholine; white circles, polyamine supplement; inverted black triangles, antioxidant supplement; white triangles, cholesterol. The data points were plotted using maximum viable cell concentrations of three independent cultures.

Figure 2 shows the normal probability plot of the fractional factorial design. In the normal plots, the ordered effects are plotted on the x-axis, and the appropriate normal % probability is plotted on the y-axis. If some of the variables affect the response, real effects will fall off the line in the plot, either high and to the right (for positive effects) or low and to the left (for negative effects) [18].

The main effect of phosphatidylcholine, located in the lower left-hand portion of the plot, was always negative with regard to cell growth, consistent with the previous assumption. Cholesterol, located in the upper right-hand of the plot, always showed a positive effect on cell growth. In the case of the polyamine supplement, a positive effect was shown in two out of the three tests. As for the antioxidant supplement, a negative effect was observed in two out of the three tests. Accordingly, among the four candidates, excluding the clearly negative candidate (phosphatidylcholine) and one possibly negative candidate (antioxidant supplement), cholesterol and polyamine supplement were chosen as active factors on cell growth.

### Determination of optimal concentrations of supplements using a statistical analysis

In order to optimize the concentration of cholesterol and polyamine supplement, 9 kinds of SFM were designed by the response surface method. Cell cultures were performed with IL-2 supplementation, as described in the Materials and Methods section.

**Figure 3:**
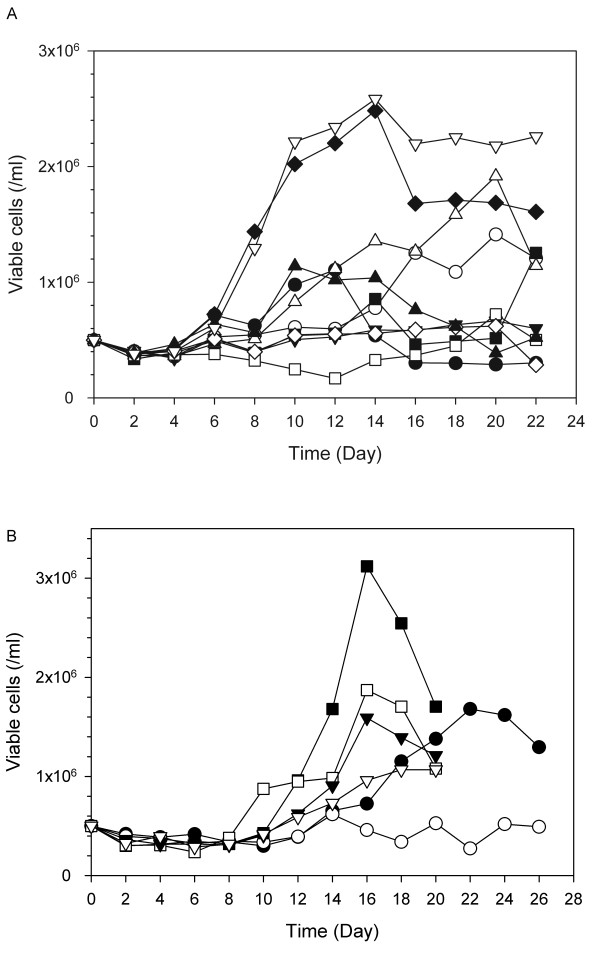
**Response surface design for determination of optimal concentrations of supplements**. A. SFM with various combinations of cholesterol and polyamine supplement designed by the response surface method. B. Response surface graph for the effect of cholesterol and polyamine supplement on maximum viable cell concentration.

Figure 3A shows various combinations of cholesterol and polyamine supplement with a range of 0 and 4× in these SFM. Excessively high concentrations of cholesterol and polyamine supplement did not promote cell growth in the preliminary cultivation (data not shown). PBMCs from the three different donors were cultivated independently in these media for approximately three weeks. As summarized in Table 4, the highest maximum viable cell concentration was always achieved in SFM* #4. Compared to the control SFM (SFM* #2), an approximate 1.4-fold increase in maximum viable cell concentration was achieved in SFM* #4. The results of the analysis of the response surface design using Design-Expert^® ^led to the optimized composition of SFM shown in Figure 3B. Thus, the preferred SFM formulation is the basal SFM supplemented with 3.3× of cholesterol (13.2 mg/l) and 0.1× of polyamine supplement (Sigma, #P8483).

**Table 4:** Maximum viable cell concentrations in 9 SFM designed by the response surface method.

SFM	Maximum viable cell concentration (10^5 ^cells/ml)		
	
	Set #1	Set #2	Set #3
SFM* #1	18.12	16.47	9.66
SFM* #2	14.49	20.97	12.87
SFM* #3	22.68	9.45	13.38
SFM* #4	23.13	23.67	17.64
SFM* #5	9.01	9.48	8.61
SFM* #6	10.83	12.48	5.21
SFM* #7	15.21	21.24	16.20
SFM* #8	9.39	6.78	9.04
SFM* #9	12.78	13.20	7.11

Concentrations of cholesterol and polyamine supplement in each SFM are shown in Figure 3A. Three sets of cultures were performed independently with PBMCs obtained from the three different donors. The seeding density was 5 × 10^5 ^cells/ml.

To confirm cell growth in the optimized SFM, PBMCs were cultivated with IL-2 supplementation in 10 kinds of SFM, including the optimized SFM and the 9 designed SFM shown in Figure 3A.

**Figure 4:**
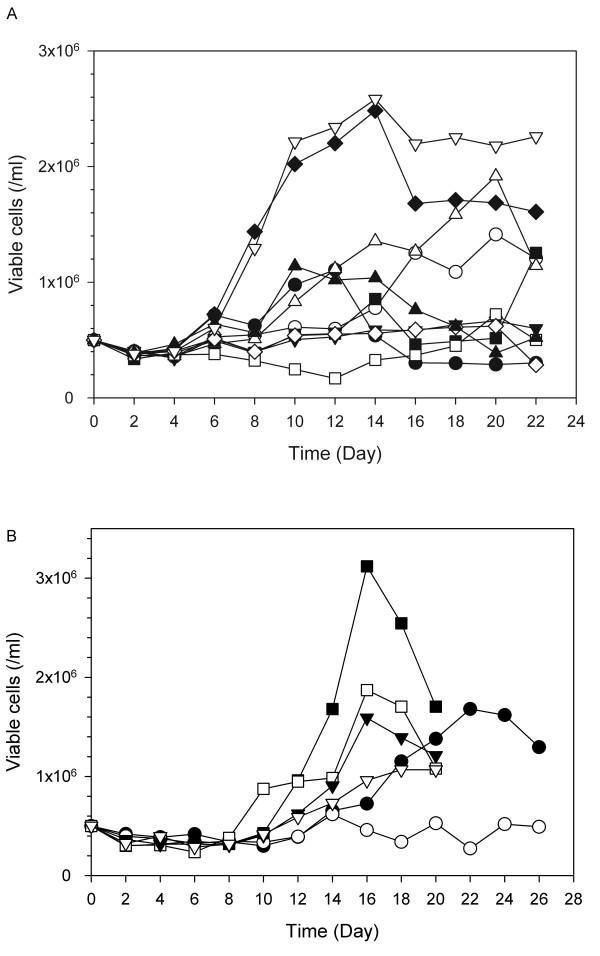
**Growth profiles of cells cultured in an optimized SFM**. A. Comparison with SFM* shown in Figure 3A. Black circles, SFM* #1; white circles, SFM* #2; inverted black triangles, SFM* #3; white triangles, SFM* #4; black squares, SFM* #5; white squares, SFM* #6; black diamonds, SFM* #7; white diamonds, SFM* #8; black triangles, SFM* #9; inverted white triangles, optimized SFM. This figure shows one representative growth profile among the three independent cultures. B. Comparison with SCM. Three sets of cultures were performed independently with PBMCs obtained from the three different donors. Symbol indicates each set. Black symbols represent cells grown in optimized SFM, and white symbols represent cells grown in SCM.

Figure 4A shows typical cell growth profiles of PBMCs in SFM. Cell growth in the optimized SFM was compared with the other SFM designed by the response surface method. In all three independent cultures of PBMCs isolated from the three different donors, cell growth in the optimized SFM was always better than that in any other medium. Growth performance in the optimized SFM was also compared with that in SCM. As shown in Figure 4B, growth performance of PBMCs in the optimized SFM depended on the donor. However, cell growth in the optimized SFM was always better than that in SCM.

### Phenotypical analysis of cells cultured in developed SFM

PBMCs comprise monocytes and lymphocytes including T cells (CD4^+ ^and CD8^+^), B cells, and NK cells. To determine the possible changes in cell population of PBMCs cultured in the developed SFM with IL-2 supplementation, subsets of the population at the maximum viable cell concentration in SFM as well as SCM, as shown in Figure 4B, were analyzed by flow cytometry with fluorochrome-conjugated monoclonal antibodies (CD14 to monocytes, CD3 to CD4^+ ^and CD8^+ ^T cells, CD8 to CD8^+ ^T cells, and CD19 to B cells).

Initially, the major cell population was T lymphocytes, while 13-27% of PBMCs were monocytes and B cells. Regardless of the culture media, most of the viable cells, after cultivation with IL-2 supplementation, were T lymphocytes, while monocytes and B lymphocytes were not detected. The percentage of CD3^+ ^T lymphocyte population was similar between SFM and SCM (*p *= 0.22, n = 3). The results of the phenotypical analysis are summarized in Table 5.

**Table 5:** Phenotypical analysis of cells cultured in developed SFM and SCM.

Set #1	PBMCs (%)^a^	SFM (%)^b^	SCM (%)
CD14	5.21	-^c^	-
CD19	20.63	-	-
CD3	58.80	81.34	83.72
CD8	20.42	30.50	29.86

**Set #2**	**PBMCs (%)**	**SFM (%)**	**SCM (%)**

CD14	6.66	-	-
CD19	19.97	-	-
CD3	62.99	84.32	88.83
CD8	19.56	18.6	11.23

**Set #3**	**PBMCs (%)**	**SFM (%)**	**SCM (%)**

CD14	1.55	-	-
CD19	11.53	-	-
CD3	64.58	78.34	90.82
CD8	19.32	28.58	6.80

^a ^Freshly thawed PBMCs, before cultivation, were analyzed.

^b ^Cell samples in the three independent cultures were taken at a maximum viable cell concentration (refer to Figure 4B).

^c ^not detected.

### Functional assays of cells cultured in developed SFM

To determine the effector function of CTLs cultivated in the developed SFM, antigen-specific CTLs were first generated from PBMCs, cultured in both the developed SFM and SCM, by stimulation of peptide-presenting DCs. The effector function of CTLs was then characterized by a cytotoxicity assay and an ELISpot assay. Functional assays were duplicated independently.

**Figure 5:**
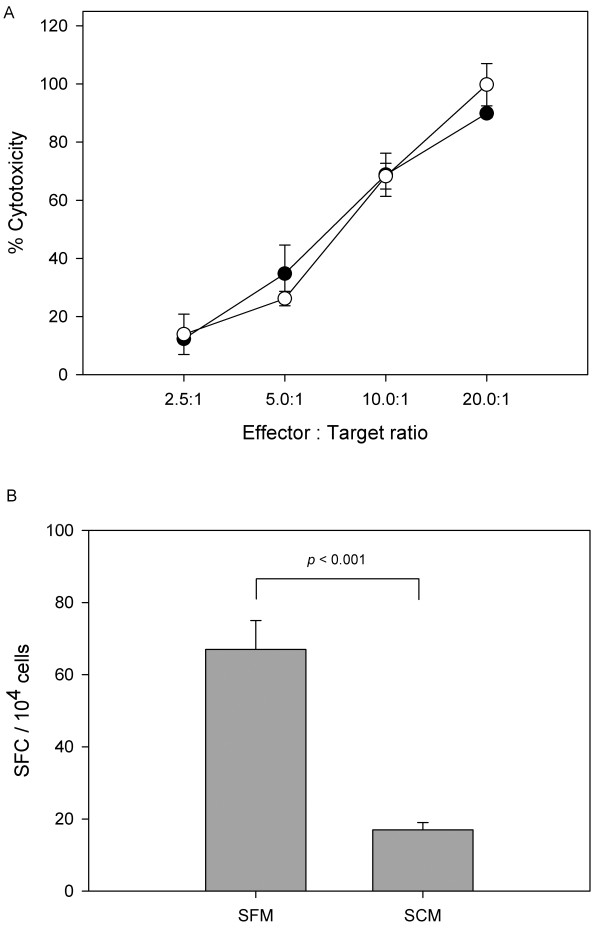
**Functional assays of CMV-specific CTLs generated from PBMCs cultured in the developed SFM and SCM**. A. Cytotoxicity of CMV-specific CTLs generated from PBMCs cultured in the developed SFM (black circles) and SCM (white circles) was tested for their ability to lyse target cells. B. CMV-specific responses obtained by ELISpot using the developed SFM and SCM as culture media. Functional assays were duplicated independently and each experiment was carried out in triplicates. Error bar represents the standard deviation (n = 3).

Figure 5A shows the % cytotoxicity of antigen-specific CTLs. When 1 × 10^5 ^cells/ml of target cells were cultivated with effector cells at a ratio of 1:20, the % cytotoxicity of antigen-specific CTLs generated from PBMCs that had been cultured in the developed SFM, reached approximately 90%; with dilution of these ratios to 1:10, 1:5, and 1:2.5, the % cytotoxicity decreased to 69%, 35%, and 12%, respectively. Considering the standard deviations generated between replicated wells, the cytotoxicity of the cells cultured in the two media, SFM and SCM, was approxymately the same (*p *= 0.47, *p *= 0.97, *p *= 0.27, and *p *= 0.74 at each ratio).

Figure 5B shows the results of the ELISpot assay. Unlike the cytotoxicity assay, the results of the ELISpot assay differed to a certain degree according to the medium in which the PBMCs were grown. In the case of PBMCs cultured in the developed SFM, an average of 67 spot-forming cells (SFC)/10^4 ^effector cells were observed after subtracting background spots, whereas in the case of PBMCs cultured in the SCM, an average of 17 SFC/10^4 ^effector cells were shown. SFC in SCM decreased to one-fourth of that seen in SFM (*p *< 0.001). According to a previous study [19], use of SFM should enhance (2.4-fold median increase) detection sensitivity in the ELISpot assay, which corresponds to the results of this study. Taken together, the use of developed SFM to culture PBMCs was advantageous, considering the cellular function as well as growth profiles.

## Discussion

In developing SFM for *in vitro *expansion of human CTLs, a limitation of cell number from the same donor thwarted the employment of a full factorial design. In this study, a simple approach to screen media supplements that enhance the growth of T lymphocytes was described. The approach combined elements of statistical experimental design with maximum cell concentration to identify potent growth responses of T lymphocytes. The use of normal probability plots confirmed the experimental results in a statistical sense which provided a simple means of analyzing and prioritizing the consequences of our experimental results.

Although serum has previously been replaced by several supplements in lymphocyte cultures [8], the SFM developed in this study showed a more enhanced effect on the growth of lymphocytes. We adopted a basal SFM with the serum-free components listed in Table 1. Transferrin, a widely used but expensive growth factor, was also successfully replaced by ferric citrate which could deliver iron to cells. In addition, 2-mercaptoethanol and 1-thioglycerol were used because they were reported as positive factors on proliferation and activation of lymphocytes [9].

Among four candidates chosen to further enhance the growth of lymphocytes, cholesterol and polyamine supplement were selected as active factors on cell growth by analysis of Design-Expert^®^. Cholesterol, one of the major lipid constituents in serum, is a major lipid component of the plasma membrane in most cells, and polyamines including putrescine and spermidine are commonly used to supplement SFM for a variety of cell types [20-22]. The optimal concentrations of these two supplements were 3.3× of cholesterol and 0.1× of polyamine supplement. The optimal concentration of polyamine supplement was quite low, compared to that of cholesterol. In three independent cultures, cholesterol always showed a positive effect on cell growth. However, in the case of polyamine supplement, a negative effect on growth was observed in one case. Polyamine supplement, similar to antioxidant supplement, which showed inconsistent responses in repeated experiments, displayed a marginal effect on cell growth.

On the other hand, although being universally used in SFM as a lipid source, phosphatidylcholine showed a significantly negative effect on the growth of lymphocytes. Phosphatidylcholine is one of the phospholipids that constitute serum, and it is known as an active factor on cell growth [12,23]. The effect of phosphatidylcholine, however, could be affected by the kinds of cells cultured and the concentration of phosphatidylcholine used [24,25]. In addition, the effects of phosphatidylcholine on cell growth were influenced by the lipids present in serum [26]. Therefore, it is assumed that lymphocyte culture can be negatively affected by phosphatidylcholine, especially when fatty acids are supplemented as a lipid replacement.

For the rapid generation of large numbers of CTLs, different strategies have been employed, each having respective, and debatable, pros and cons. In some cases, CD8^+ ^T cells are initially separated from PBMCs, and the process of expansion is then completed [27]. Such a strategy would yield a greater number of CTLs at the end of the culture. However, in this study, since the focus was on developing a SFM, culture strategy was not emphasized.

As expected, growth profiles of PBMCs, which are not a continuous cell line, varied depending on the donors. In some cases, when cells were cultured in SCM, they did not grow (Figure 4B). Furthermore, with regard to the % of CD8^+ ^lymphocytes in the cultured cells, variation between donors in SCM was more significant than that in SFM (Table 5). Therefore, use of SFM can reduce the variation that can be generated from many causes during the culture period. Furthermore, cytotoxicity and ELISpot assays revealed that the effector function of CTLs cultivated in the developed SFM was retained or even better than that in SCM.

## Conclusions

SFM with cholesterol and polyamine supplement for human lymphocyte culture was developed efficiently using a statistical method. This SFM provides better or at least equivalent performance with respect to cell growth, variation in cell population, and cytotoxicity, compared to SCM.

## Methods

### Medium composition and preparation

A basal SFM, which was developed in our laboratory, was based on RPMI1640 medium (Invitrogen, Grand Island, NY) containing 2 g/l NaHCO_3 _(Sigma, St. Louis, MO). All supplements used in the basal SFM, unless otherwise specified, were purchased from Sigma and their concentrations are given in Table 1. Insulin and ferric citrate were prepared in 1 M acetic acid and boiling water, respectively. Fatty acids such as linoleic acid, oleic acid, and palmitic acid were first dissolved in ethanol and then diluted in culture medium. RPMI1640 non-essential amino acids (Sigma, #R7131) and vitamins solution (Sigma, #R7256) were added as 1× concentration in culture medium before use. Other supplements were dissolved in water before addition to the culture medium.

Fetal bovine serum (FBS) used in serum-containing medium (SCM) was purchased from Invitrogen, and a commercial SFM, X-VIVO15, was purchased from Lonza (Walkersville, MD). Interleukin-2 (IL-2) (Millipore, Bedford, MA), which was added into the culture media as a growth factor, was reconstituted in 100 mM acetic acid.

### Potential components for growth enhancement

Four supplements were chosen according to their general function in cell culture. The supplements were (1) phosphatidylcholine (5 mg/l), (2) polyamine supplement (#P8483), (3) antioxidant supplement (#A1345), and (4) cholesterol (4 mg/l) (all from Sigma). Polyamine supplement and antioxidant supplement were supplied as 1000× concentrates. Phosphatidylcholine and cholesterol were first dissolved in ethanol and then diluted in the culture medium before use.

### Experimental design and statistical analysis

An experimental design was applied using Design-Expert^® ^software (version 7.1.2, Stat-Ease, Inc., MN) employing one-half fraction of the two-level factorial design with 4 factors (2^4-1^) involving 8 combinations of all factors (Table 2). The 4 supplements were screened using two-level fractional factorial designs to estimate their effect on maximum cell concentration. After selecting two positive determinants on growth, 9 kinds of SFM were determined by response surface designs with range of these supplement concentrations between 0 and 4×. Design-Expert^® ^provided a fractional factorial matrix, a normal probability plot, a response surface matrix, and a contour plot of the predicted elongation values of maximum cell concentration. It was then possible to find the optimal concentrations of two positive supplements for cell growth. The response surface designs were analyzed by fitting a quadratic model. The model adequacy was confirmed using analysis of variance (ANOVA).

### *In vitro *lymphocyte culture

Peripheral blood mononuclear cells (PBMCs) were collected by apheresis from normal HLA-A0201 donors after obtaining informed consent. PBMCs were isolated from the apheresis product by Ficoll-Hypaque density gradient centrifugation (Pharmacia Biotech, Wikstrom, Sweden) and cryopreserved at -160°C in human AB^+ ^serum and RPMI1640 medium (Invitrogen) containing 10% DMSO (Sigma), as described previously [28].

After thawing, isolated PBMCs were washed twice with phosphate-buffered saline (PBS) before seeding. PBMCs (5 × 10^5 ^cells/ml) were plated into 24-well plates (Nunc, Roskilde, Denmark) containing 2 ml of culture media and then cultivated in a 5% CO_2_/air mixture, humidified at 37°C. Every two days, 1 ml of culture supernatant was gently replaced with 1 ml of the fresh medium supplemented with IL-2 (Millipore). The final IL-2 concentration in culture media was 50 U/ml. For determination of viable cell concentration, each well was sacrificed every other day. Viable cell concentration was estimated by the trypan blue dye exclusion method using a hemacytometer. Unless specified, all cell cultures were performed three times with PBMCs isolated from different donors.

### Generation of CMV-specific CTLs

To generate CMV-specific CTLs for functional assays, peptide-loaded autologous dendritic cells (DCs) were first generated as previously described [4,28]. Briefly, PBMCs were incubated for 2 h at 37°C in X-VIVO15 medium. Adherent monocytes were suspended at a concentration of 1 × 10^6 ^cells/ml in X-VIVO15 supplemented with GM-CSF (800 U/ml, Millipore) and interleukin-4 (IL-4, 1000 U/ml, Millipore). On day 2 and 4 of culture, spent medium was exchanged with fresh medium containing 1600 U/ml of GM-CSF and 1000 U/ml of IL-4. On day 5, 200 U/ml of tumor necrosis factor-α (TNF-α) (Millipore) was added for the maturation of DCs. After 48 h maturation, autologous DCs were pulsed with NLVPMVATV peptides (10 μg/ml, Peptron, Korea), HLA-A0201 restricted CMV peptide epitope, for at least 2 h and then irradiated (25 Gy).

PBMCs cultured *in vitro *using culture media supplemented with IL-2 (developed SFM or serum-containing medium (SCM): RPMI1640 supplemented with 10% FBS) were plated at a concentration of 1 × 10^6 ^cells/well in a 24-well culture plate (Nunc) with 2 ml of the corresponding culture media and directly stimulated with peptides at a concentration of 10 μg/ml (day 0) and with peptide-pulsed of autologous DCs (at a ratio of 1:10 DCs to PBMCs, day 7, day 14 and day 21 for a 4-week expansion). Every two days, 1 ml of culture supernatant was gently replaced with 1 ml of the fresh medium supplemented with IL-2 (50 U/ml, Millipore). After 24 days of cultivation, cells were subjected to cytotoxicity and ELISpot assays.

### Flow cytometry

Fresh PBMCs isolated by Ficoll-Hypaque density gradient centrifugation and 15-day cultured PBMCs were prepared for flow cytometric phenotypic analysis. Flow cytometry was performed according to standard procedures. In brief, 5 × 10^5 ^cells/tube were stained in the dark for 20 min at 4°C with FITC-labeled anti-CD3, PE-labeled anti-CD8, FITC-labeled anti-CD19 or PE-labeled anti-CD14 monoclonal antibody. All antibodies were obtained from BD Biosciences (San Diego, CA). Unstained cells were used as a negative control. After washing in PBS, cells were analyzed on a FACS LSRII (BD Biosciences) using Flowjo software (Tristar, San Carlos, CA)

### Cytotoxicity

The cytotoxic specificity was determined by lysis of target cells using a lactate dehydrogenase (LDH)-release assay (CytoTox 96^® ^non-radioactive cytotoxicity assay kit, Promega, Madison, WI) according to the manufacturer's instructions. Peptide-sensitized PBMCs were used as effector cells and peptide-presenting autologous DCs were used as target cells. Each test was repeated twice in two media (SFM and SCM). Target cells (1 × 10^5 ^cells/ml) were cultured with effector cells at various ratios of target cells to effector cells (1:20, 1:10, 1:5, and 1:2.5) in 96-well U-bottom plates (Nunc) in 100 μl of culture media for 6 h. Cells were plated in triplicates at each ratio. LDH release was quantified by measuring wavelength absorbance at 490 nm. The percentage of target cell lysis was calculated according to the following formula: [(experimental LDH release - target cells spontaneous LDH release - effector cells spontaneous LDH release) × 100]/[(target maximum LDH release - target spontaneous LDH release)].

### ELISpot assay

To determine the frequency of T lymphocytes capable of responding to a specific stimulus by secretion of IFN-γ, an ELISpot assay was carried out as described previously [29-31]. Polyvinylidene fluoride (PVDF) plates (Millipore) were coated with anti-IFN-γ monoclonal antibody (BD Biosciences) (10 μg/ml in PBS) and incubated overnight at 4°C. Plates were then washed 6 times with PBS to remove unbound antibody. After blocking with culture media (SFM or SCM) at room temperature for 1 h, peptide-sensitized PBMCs (1 × 10^4^/well) were plated in triplicates with peptide-presenting autologous DCs (1 × 10^3^/well in 100 μl of culture media. Cells cultured without activation with DCs were used as a control. After incubation at 37°C, 5% CO_2 _humidified incubator for 24 h, cells were removed from the plates by 5 washes with PBS and one wash with distilled water. PBS/T was then used for all further washing steps. Wells were incubated with 100 μl of biotinylated monoclonal anti-human IFN-γ antibody (1 μg/ml PBS/FBS 10%, BD Biosciences) for 2 h at 37°C, 5% CO_2_, to detect captured IFN-γ. After 6 washes, 100 μl of avidin-horseradish peroxidase conjugate (1:1000 dilution in PBS/FBS 10%, BD Biosciences) was added. After 1 h incubation at room temperature, wells were washed six times. Coloration was developed with 3-Amino-9-ethylcarbazole (AEC, Sigma) and the reaction was terminated after 20 min by washing the plates with distilled water. The visible spots were counted using a Stemi 2000-C dissecting microscope (Carl Zeiss, Inc., Thornwood, NJ).

## Authors' contributions

MKJ, JBL, and GML designed the research. MKJ performed all the experiments and analyzed the data. JBL supported by supplying PBMCs. JBL and GML conceived the study. MKJ and GML wrote the manuscript. All authors have read and approved the final manuscript.
